# Subjective Environmental Experiences and Women’s Breastfeeding Journeys: A Survival Analysis Using an Online Survey of UK Mothers

**DOI:** 10.3390/ijerph17217903

**Published:** 2020-10-28

**Authors:** Laura J. Brown, Sarah Myers, Abigail E. Page, Emily H. Emmott

**Affiliations:** 1 Department of International Development, London School of Economics & Political Science, London WC2A 2AE, UK; 2 Institute for Global Health, University College London, London NW3 2PF, UK; 3 UCL Anthropology, University College London, London WC1H 0BW, UK; sarah.myers@ucl.ac.uk (S.M.); emily.emmott@ucl.ac.uk (E.H.E.); 4 BirthRites Independent Max Planck Research Group, Max Planck Institute for Evolutionary Anthropology, 04103 Leipzig, Germany; 5 Department of Population Health, London School of Hygiene & Tropical Medicine, London WC1E 7HT, UK; Abigail.Page@lshtm.ac.uk

**Keywords:** UK, breastfeeding, subjective environmental experiences, physical environment, social environment, social support, crime, littering

## Abstract

Local physical and social environmental factors are important drivers of human health and behaviour. Environmental perception has been linked with both reproduction and parenting, but links between subjective environmental experiences and breastfeeding remain unclear. Using retrospective data from an online survey of UK mothers of children aged 0–24 months, Cox-Aalen survival models test whether negative subjective environmental experiences negatively correlated with any and exclusive breastfeeding (max *n* = 473). Matching predictions, hazards of stopping any breastfeeding were increased, albeit non-significantly, across the five environmental measures (HR: 1.05–1.26) Hazards for stopping exclusive breastfeeding were however (non-significantly) *reduced* (HR: 0.65–0.87). Score processes found no significant time-varying effects. However, estimated cumulative coefficient graphs showed that the first few weeks postpartum were most susceptible to environmental influences and that contrary to our predictions, mothers with worse subjective environmental experiences were *less* likely to stop breastfeeding at this time. In addition, the hazard of stopping exclusive breastfeeding *declined* over time for mothers who thought that littering was a problem. The predicted increased hazards of stopping breastfeeding were only evident in the later stages of any breastfeeding and only for mothers who reported littering as a problem or that people tended not to know each other. Perceived harsher physical and social environmental conditions are assumed to deter women from breastfeeding, but this may not always be the case. Women’s hazards of stopping breastfeeding change over time and there may be particular timepoints in their breastfeeding journeys where subjective environmental experiences play a role.

## 1. Introduction

The UK has some of the lowest breastfeeding rates in the world. Whilst many UK mothers initiate breastfeeding, continuation rates remain low [1,2]. With breastfeeding providing a myriad of health benefits for both mothers and infants, this is a key public health problem [3,4], and one that does not just fall on mothers to solve. Infant feeding decisions are complex and multifactorial, and there are many layers of influence from family attitudes and social norms to economic and policy drivers [4,5,6,7,8]. Furthermore, a growing body of research suggests that physical and social environmental factors play a key role in women’s infant feeding journeys [9,10,11,12,13,14].

Local environmental factors have well-established links with a range of health outcomes, from chronic diseases [15] and the aging process [16], to health behaviours [17], mental health outcomes [18], and social well-being [19]. Harsher environments as indexed by greater neighbourhood deprivation, higher levels of crime, exposure to environmental hazards, and other place-based stressors have also been shown to predict reproductive and parenting behaviour. Mothers in harsher environments have been reported to have earlier first births, more births and be at greater risk of preterm deliveries, small for gestational age and lower birthweight infants [13,20,21,22,23]. Further, mothers have been shown to exhibit lower maternal warmth and harsher/more controlling parenting practices in more dangerous environments (i.e., those perceived to have more violence, crime and social disorder) [24,25]. Linked to health outcomes for mother and infant, connected with women’s fertility and a key component of parenting, it is therefore not surprising that breastfeeding has also been shown to be environmentally influenced.

How environmental influences are conceptualised and measured varies across studies. The Index of Multiple Deprivation (IMD), a summary score based on several environmental domains and available at electoral ward level (or equivalently sized geographies) [26] is conceptualised as area-based deprivation, and is a commonly used metric in environment-breastfeeding research. Findings have however been mixed, with greater deprivation associated with shorter breastfeeding durations in some studies [12,13,27] but not others [28]. Possible reasons for such mixed results include the overlooking of individual subjective environmental experiences [29,30] given the focus on objective or independent neighborhood assessments [9,31,32]. Further, composite measures like the IMD combine both physical and social environmental factors into a summary score, making it difficult to discern specific pathways or mechanisms of influence [32].

Human environments are complex and comprise a multitude of threats and resources, the combination of which varies greatly across contexts. Physical aspects of the environment comprise both natural and built components [33] and include environmental hazards such as pollution [34], but also positive aspects like the availability of green spaces [35,36] and general perceptions of how “nice” an area is [9,37]. On the other hand, social environmental factors include aspects related to community cohesion and safety such as neighbour friendliness and levels of crime [14,38]. Physical and social factors are however often interrelated, with for example, physical incivilities such as littering leading to increased fear of crime [39].

Some environmental aspects are more perceivable than others. Previous research has shown that visually non-detectable levels of pollution in air and water, i.e., physical aspects of the environment, are linked with breastfeeding outcomes [10], suggesting that some environmental threats may impact the physiology of lactation directly [40]. This is in contrast to more salient and perceivable environmental cues, such as the behaviour of others, i.e., the social environment, which instead may affect breastfeeding through psychological or cognitive pathways, i.e., mothers perceiving and processing information (consciously or unconsciously) and then altering their behaviour accordingly.

Environmental perception therefore provides an important potential explanation for how environmental threats and resources are translated into behavioural changes. As already discussed, local environmental conditions have been shown to predict a range of reproductive behaviours and outcomes [13,20,21,22,23]. Other studies have suggested that environmental perception may be driving some of these associations [37,41]. For example, women who perceive their environments unfavourably are more likely to start reproducing at younger ages [13,37] and to have lower birth weight infants [13,31]. While increasing evidence suggests subjective environmental experiences influence reproductive behaviours and outcomes, the relationship between environmental perception and breastfeeding duration remains relatively underexplored. The one study we know of that has looked at this found only weak evidence for a link between subjective environmental quality and duration of any breastfeeding [9]. That study combined a mix of subjective environmental experiences into a summary score, making it difficult to identify which aspects of the physical and/or social environment may be relevant. Furthermore, only breastfeeding initiation and duration of any breastfeeding (i.e., including mixed-feeding) were considered, and relationships with exclusive breastfeeding remain untested. The evidence for links between environmental perception and breastfeeding therefore remains inconclusive. Understanding environmental influences on breastfeeding duration can guide interventions to improve breastfeeding rates. In addition, considering the wider environmental determinants of infant feeding can help to combat the neoliberal public health message of maternal responsibility and shift the focus away from the mother [42]. A focus on environmental perception specifically provides at least two avenues for intervention—addressing the environmental threat and/or the (mis)perception. Measuring separate and specific physical and social environmental factors—rather than obscuring potential effects in composite measures—is necessary in order to be able to identify which environmental factors or perceptions thereof need addressing.

This study aims to test whether subjective environmental experience, as measured by responses to five environmental perception questions relating to different aspects of the physical and social environment, is associated with duration of any and exclusive breastfeeding in a UK sample of mothers. This study therefore builds on previous environmental perception research by looking at individual environmental experience factors separately in an attempt to identify potential pathways of influence and intervention. Whilst the lack of existing research means the role of environmental perception in shaping infant feeding journeys remains unestablished, existing environmental health literature suggests that harsher environmental conditions lead to worse health outcomes, fewer health-promoting behaviours, more adverse reproductive outcomes and altered parenting practices. This leads us to predict that mothers who perceive their environments less favourably will have shorter breastfeeding durations.

## 2. Materials and Methods

### 2.1. Data Collection

In this study we use data from a retrospective online survey which was developed as part of a wider project on social support and maternal experience (https://osf.io/7kb5q/) and hosted on Opinio (survey platform; http://objectplanet.com/opinio/). The survey included questions on maternal characteristics, household characteristics, infant characteristics, birth experiences, support experiences and infant feeding experiences, taking 15–20 min to complete. For more information on the survey, see https://osf.io/dbtpy/ and information published in [43].

We took an opportunistic approach and recruited participants through convenience-sampling between December 2017 and February 2018. While convenience-sampling is likely to introduce recruitment bias, it is cost and time efficient [44]. Survey adverts were posted on social networking sites (Twitter and Facebook), as well as a forum-based parenting website (Netmums). The Facebook groups were diverse, including parenting groups, infant feeding support groups, and second-hand baby-item groups. We specifically targeted local Facebook groups around the UK in an attempt to diversify our sample. However, despite these efforts it must be noted our sample is not representative of the broader population of UK mothers and our analyses are therefore only exploratory in nature; any extrapolation of these results beyond this sample should be done with caution.

On the survey landing page, participants were informed that there would be some questions about infant feeding, with an explicit statement that it did not matter whether infants were breastfed or formula fed. Multiple-entries were prevented using IP-address checks. Ethical approval for the survey was obtained from the UCL Research Ethics Committee (ref: 11479/001).

### 2.2. Data Cleaning

In total 883 women accessed the online survey by early 2018. Of those 883, 74 (8.4%) started the survey but did not go on to fill out any further details. Of those that completed the majority of the survey, 757 (93.6%) resided in the UK, while 739 had children born in the UK (91.3%). Those not based in the UK were removed from the sample due to ineligibility. Further, six individuals were removed from the sample as their children were older than two years when the survey was taken. This left a total eligible sample of 733 individuals.

### 2.3. Breastfeeding Outcomes

85.4% of the eligible sample (*n* = 626) provided a measure of breastfeeding initiation and/or duration. Of those women, 43 reported they never initiated breastfeeding (6.9%), leaving a total maximum sample size of 583 for the duration of breastfeeding analysis. Breastfeeding duration was measured in weeks and both duration of any and of exclusive breastfeeding were available. Estimates in days were divided by 7 to give weeks and the age of the child was used for women who reported that they were still breastfeeding. For any breastfeeding, durations were available for 579 women. For exclusive breastfeeding, 560 mothers provided an estimate of at least 1 day. We removed cases where mothers breastfed but never exclusively (*n* = 23). Due to apparent misunderstanding of the exclusive duration questions, we restricted the sample to those who were either still breastfeeding and reported having stopped exclusive breastfeeding or those who reported any breastfeeding for longer than they exclusively breastfed, suggesting they had understood the question correctly (removing a further 141 cases). As exclusive breastfeeding is recommended to just 6 months [1,45] we also dropped cases where estimated durations were higher than 7 months (*n* = 49) as these were likely erroneous. This left a maximum sample size of 626, 579 and 351 for initiation, any breastfeeding and exclusive breastfeeding analyses, respectively.

### 2.4. Subjective Environmental Experiences

The analytical sample was restricted to those who provided a yes or no response to at least one of five environmental questions (excluding 85 individuals), which reduced the maximum sample size to 541 observations. The questions were: 1. “My area is a nice place to live” (Yes/No); 2. “Crime is a problem in my area” (No/Yes); 3. “Littering and rubbish is a problem in my area” (No/Yes); 4. “People tend to know each other in my area” (Yes/No); 5. “In general, people help each other out in my area” (Yes/No).

### 2.5. Covariates

Our initial plan was to include several infant and maternal characteristics known to be important for predicting breastfeeding outcomes: birthweight [46], maternal age [47], parity [27], partnership status [48] and ethnicity [49]. However, due to small sample size, low variation and a lack of bivariate associations (see Section 3.1 below), we decided to adjust only for maternal age and parity in our first set of models (presenting just descriptives for the other characteristics). In our second set of models we also controlled for maternal education, a proxy for socioeconomic status (SES) which is a key driver of breastfeeding outcomes [2,12,50,51,52,53]. GCSEs and A-levels (i.e., middle and high-school level qualifications) were combined into one category due to small cell sizes and “Other” was recoded as missing. We did not adjust for other aspects of socioeconomic status to avoid issues associated with collinearity and power reduction, but present the descriptives for annual income, education, partner’s employment and financial situation for interest. Restricting the sample to complete cases for maternal age, parity and education reduced the sample to 525 cases. Running multi-level analyses meant further restricting the data to those who had provided an answer to the region questions, thereby reducing the sample down further to 512 observations, of whom 32 reported never initiating breastfeeding, leaving a maximum usable sample size of 480 and 293 for duration of any and exclusive breastfeeding, respectively.

Of these 512 mothers, responses were coded as NA/missing if they did not answer the partner’s employment (*n* = 11), or birthweight (*n* = 9) questions, or answered “Don’t know” (*n* = 21), “Prefer not to say” (*n* = 5) or didn’t answer (*n* = 2) the income question, or answered “Prefer not to say” (*n* = 2) or didn’t answer (*n* = 2) the financial situation question.

Annual income was split into high earners “£50,000 and above” or low earners “Under £50,000” as it was not clear if respondents provided take-home or pre-tax annual income and this was not equivalised income (i.e., we couldn’t be confident that, for example, £20,000 was meaningfully different from £30,000). Several other covariate categories were collapsed due to small cell sizes. “Looking for work” and “Not looking for work” were collapsed into “Unemployed” for partner’s employment status and “Finding it very difficult” and “Finding it quite difficult” were collapsed into “Finding it difficult” for financial situation. All non-White ethnicities were collapsed into “Other” for ethnicity (since our sample was overwhelming White leaving little variance across other categories). Given that multiparous mothers are more confident and experienced with breastfeeding [54], we categorised parity into “1st birth” and “2nd birth or higher” (collapsing “2”, “3”, “4” and “5”). Birthweights were sense-checked and recoded where obvious mistakes had been made (i.e., unit omitted, wrong unit listed) before being categorised into low (<2500 g) and normal (≥2500 g). Most respondents did not answer the multiple birth question and so we were unable to reliably exclude multiple births from the analyses, though we expect these to be low in number.

### 2.6. Data Analysis

As an initial descriptive analysis, we used Pearson’s Chi-squared test with Yates’ continuity correction and Fisher’s Exact Test where there were small cell sizes to compare probabilities of initiating breastfeeding across different environmental experiences, maternal and infant characteristics and socioeconomic statuses. We used Wilcoxon Rank Sum tests (2 groups) and Kruskal Wallis tests (3+ groups) to compare median breastfeeding durations across the same variables (as distributions of both any and exclusive breastfeeding durations were right/positively-skewed). Post-hoc Dunn tests were used to test which categories had significantly different medians.

It was predicted that there would be a diminishing probability of maintaining breastfeeding over time. However, visual inspection of QQ plots and the Kolmogorov-Smirnov tests revealed that neither the duration of any (D = 0.819, *p* < 0.001) nor exclusive (D = 0.608, *p* < 0.001) breastfeeding followed the predicted parametric Weibull distribution. The distributions were actually bimodal with any breastfeeding peaking around 0–10 and 30–40 weeks and exclusive breastfeeding peaking around 0–5 and 25–30 weeks.

Cox proportional hazards models were then pursued but some of the main predictors and covariates did not satisfy the proportional hazards assumption. We therefore used Cox-Aalen models which allowed for time-varying effects (using the *timecox* option of the *timereg* package in R [55]). Time–varying effects are presented as non-standard cumulative estimates (i.e., the hazard rate as the cumulative log of the hazard rate and the effects of non-proportional hazards covariates as the cumulative effect of the covariate over time) which have different interpretations to standard de-cumulated estimates. This limitation is offset by the package’s advantages. Firstly, the *timecox* package enables controlling for clustering at the area-level, which is not possible with the standard Cox proportional hazards model. Secondly, *timecox* permits testing whether parameters have significant effects on survival outcomes (with the Supremum-test) and additionally whether these effects are time-invariant (with the Kolmogorov-Smirnov and Cramer von Mises score processes tests) under the non-proportional hazards setting [55,56,57].

We ran two sets of main models for each environmental predictor. The first set of models (M1) adjusted for maternal age and parity and for clustering at the region level and the second set (M2) additionally for education to test whether socioeconomic status confounded the relationship between subjective environmental experiences and breastfeeding outcomes. Exclusive breastfeeding survival models were capped at 6 months. Due to differing missingness on our exposure variables, analytical sample sizes for our survival analyses ranged from 409 to 473 and 250 to 290 for duration of any and exclusive breastfeeding, respectively.

We used proportionality tests in separate Cox proportional hazards models and Aalen’s additive hazards models to decide which covariates should be set as constant (i.e., proportional hazards, multiplicative effect) and which should be set as time-varying (i.e., non-proportional, additive effect) in our main Cox-Aalen models testing for the effects of the five environmental experience questions on breastfeeding duration. These diagnostic models were fully adjusted for maternal age, parity and education but excluded the environmental indicators. Overall the results of the different tests for proportionality indicated that parity should be set as constant and age should be set as time-varying for both any and exclusive breastfeeding, whilst education should be set as time-varying for any breastfeeding and constant for exclusive breastfeeding (see Table A1 in the Appendix A).

## 3. Results

### 3.1. Sample Characteristics

93.8% of the mothers in our restricted sample initiated breastfeeding (*n* = 480) and with the exclusions described above, duration of any and exclusive breastfeeding were determinable for 100% and 61% of these mothers, respectively. The median durations of which were 31.6 weeks (Interquartile range [IQR] 42.8 weeks) and 22.1 weeks (IQR 23.7 weeks). This highlights that our sample had very high levels of breastfeeding initiation compared to the UK average which was reported at 81% in 2010 [2]. Sample characteristics are shown in Table 1.

As a reminder, our sample was restricted to mothers who answered at least one of the subjective environmental experience questions, and provided area, age, parity and education information. Within this restricted sample, missingness on environmental information ranged from 1.6% (“My area is a nice place to live”) to 14.6% (“Crime is a problem in my area”). The majority of mothers reported favourable environmental experiences: only 4.3% thought that their area wasn’t a nice place to live, 11.5% thought that crime was a problem in their area, 20.3% thought that littering and rubbish was a problem, 20.3% thought that people didn’t tend to know each other in their area and 15% thought that people did not help each other out. It is important to note here that in some instances we had very few cases of unfavourable environmental experiences (e.g., only 22 mothers thought that their area was not a nice place to live). Results should therefore be interpreted with caution. The vast majority (88.5%) of mothers lived in England.

Almost half of the sample was aged between 30–34 years, 95.7% were White, 97.9% had partners, 61.1% had just the one child and 94.3% had normal birthweight infants. This was a high SES sample, with 81.6% educated to degree level or higher, 95.5% having partners who were employed, 54.3% having an annual household income of £50,000 and above, 44.9% “doing alright” financially and 28.1% “living comfortably”.

None of the environmental experience indicators were significantly associated with breastfeeding initiation or duration in bivariate analyses. Associations with breastfeeding initiation were however mostly in the predicted direction, with mothers with worse subjective environmental experiences having lower rates; the exception was mothers who disagreed that people tended to know each other in their area having higher initiation rates (95.9%) than those who agreed (93.6%, *p* = 0.470). Although there were no significant differences across environmental experience categories, average breastfeeding durations were mostly shorter for mothers with worse environmental experiences. Exceptions were mothers who agreed that crime was a problem in their area having *longer* durations of any breastfeeding (33.8 weeks [IQR 44.2] versus 31.0 weeks [IQR 42.4], *p* = 0.331) and the same average duration of exclusive breastfeeding than those who disagreed (22.1 weeks [IQR 24.6] versus 22.1 weeks [IQR 19.6], *p* = 0.537). Similarly, durations of exclusive breastfeeding were the same for mothers who did and didn’t think that littering and rubbish was a problem in their area (22.1 [IQR 19.6] versus 22.1 [IQR 24.6], *p* = 0.179).

Region was not significantly associated with breastfeeding, but there was some geographical variation with South West England having the lowest initiation rate (76.2%), whilst the West Midlands had the lowest average durations of both any (16.5 weeks [IQR 40.6]) and exclusive breastfeeding (3 weeks [IQR 40.6]). Maternal age was the only maternal or infant characteristic significantly associated with breastfeeding outcomes, and only with initiation. The proportion of respondents who initiated breastfeeding increased across age categories (*p* = 0.005), although the oldest age group had lower levels than those aged 35–39 years (91.7% versus 98.3%). Parity was associated with duration of exclusive breastfeeding but only at the 10% level, with mothers with two or more children exclusively breastfeeding for longer on average than those with just one child (26.6 weeks [IQR 22.1] versus 18 weeks [IQR 24.6], *p* = 0.083).

Education was the only socioeconomic status indicator to be significantly associated with breastfeeding outcomes. Education had a dose-response association with initiation and duration of any breastfeeding: mothers with GCSEs or AS/A-levels, graduate qualifications and postgraduate qualifications had a 89.4%, 90.4% and 98.6% chance of initiating breastfeeding, respectively (*p* < 0.001), and their median durations of any breastfeeding were 19.5 (IQR 36.5), 27.9 (IQR 35.9), and 38.7 (IQR 41.0) weeks, respectively (*p* < 0.001). Post-hoc Dunn tests showed that only mothers with GCSEs or AS/A-levels versus graduate qualifications (*p* < 0.001) and mothers with graduate qualifications versus postgraduate qualifications (*p* = 0.006) had significantly different median durations of any breastfeeding. Median durations of exclusive breastfeeding were similar for mothers with graduate (26.6 weeks [QR 23.6]) and postgraduate qualifications (26.3 weeks [IQR 22.6]), with post-hoc Dunn tests confirming that the only significant differences were between mothers with GCSEs or AS/A-levels (8.0 weeks [IQR 25.1]) and those with postgraduate qualifications (*p* = 0.003).

### 3.2. Model Results

The results of the Cox-Aalen models are shown in Table 2 and Table 3 for any and exclusive breastfeeding, respectively. These tables show results for models controlling for just age and parity (M1) and additionally education (M2), and provide hazard ratios for when environmental experiences were set as constant effects and Supremum-tests of significance and Kolmogorov-Smirnov and Cramer von Mises tests for time-varying effects when environmental experiences were set as time-varying.

**Figure 1:**
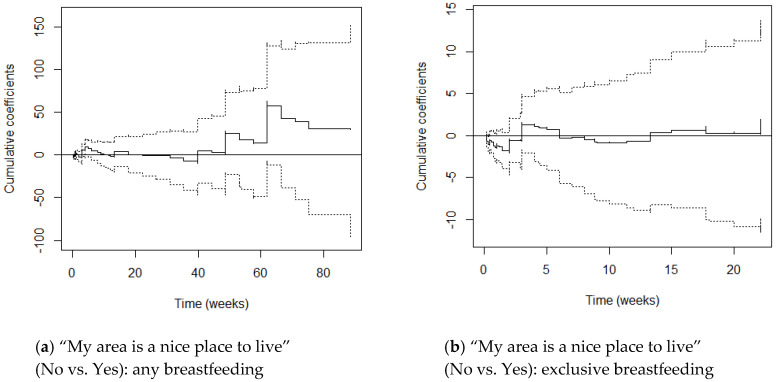
Cox-Aalen model results for “My area is a nice place to live” (No vs. Yes) for any (**a**) and exclusive breastfeeding (**b**). Main effect set as time-varying, and models adjusted for maternal age, parity and education (M2). Estimated cumulative coefficients (solid line) with 95% pointwise confidence intervals (dotted lines).

**Figure 2:**
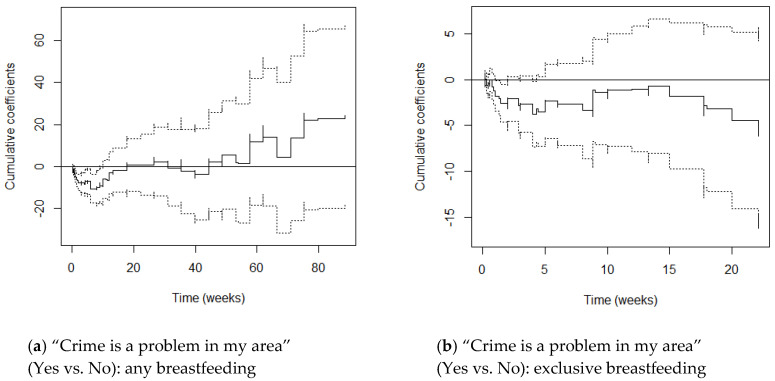
Cox-Aalen model results for “Crime is a problem in my area” (Yes vs. No) for any (**a**) and exclusive breastfeeding (**b**). Main effect set as time-varying, and models adjusted for maternal age, parity and education (M2). Estimated cumulative coefficients (solid line) with 95% pointwise confidence intervals (dotted lines).

**Figure 3:**
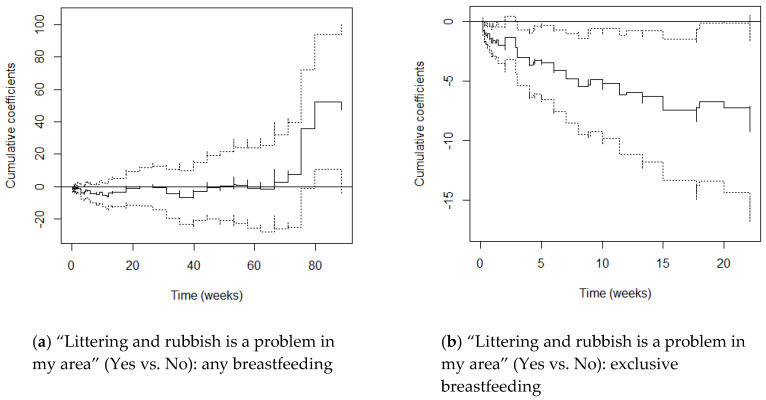
Cox-Aalen model results for “Littering and rubbish is a problem in my area” (Yes vs. No) for any (**a**) and exclusive breastfeeding (**b**). Main effect set as time-varying, and models adjusted for maternal age, parity and education (M2). Estimated cumulative coefficients (solid line) with 95% pointwise confidence intervals (dotted lines).

**Figure 4:**
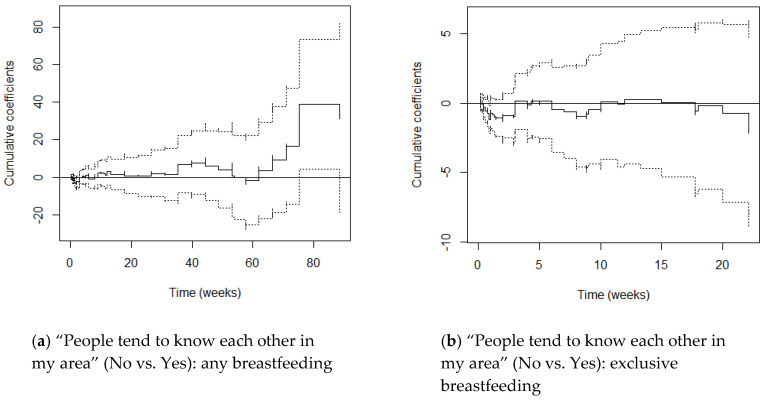
Cox-Aalen model results for “People tend to know each other in my area” (No vs. Yes) for any (**a**) and exclusive breastfeeding (**b**). Main effect set as time-varying, and models adjusted for maternal age, parity and education (M2). Estimated cumulative coefficients (solid line) with 95% pointwise confidence intervals (dotted lines).

**Figure 5:**
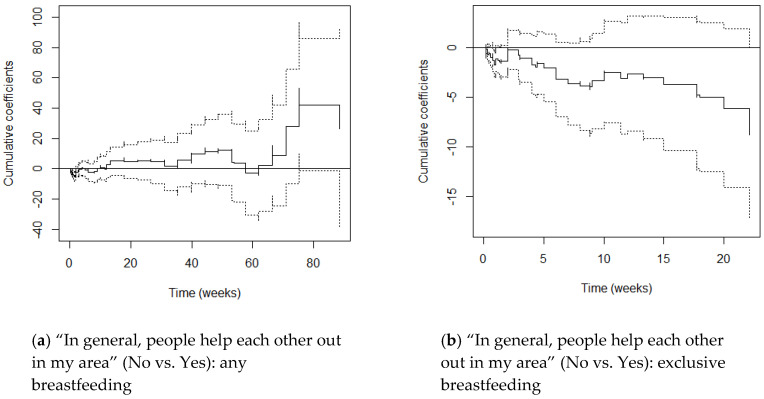
Cox-Aalen model results for “In general, people help each other out in my area” (No vs. Yes) for any (**a**) and exclusive breastfeeding (**b**). Main effect set as time-varying, and models adjusted for maternal age, parity and education (M2). Estimated cumulative coefficients (solid line) with 95% pointwise confidence intervals (dotted lines).

**Figure A1:**
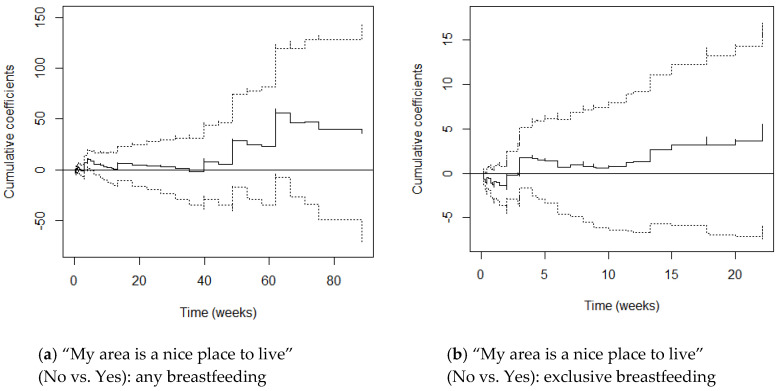
Cox-Aalen model results for “My area is a nice place to live” (No vs. Yes) for any (**a**) and exclusive breastfeeding (**b**). Main effect set as time-varying, and models adjusted for maternal age and parity (M1). Estimated cumulative coefficients (solid line) with 95% pointwise confidence intervals (dotted lines).

**Figure A2:**
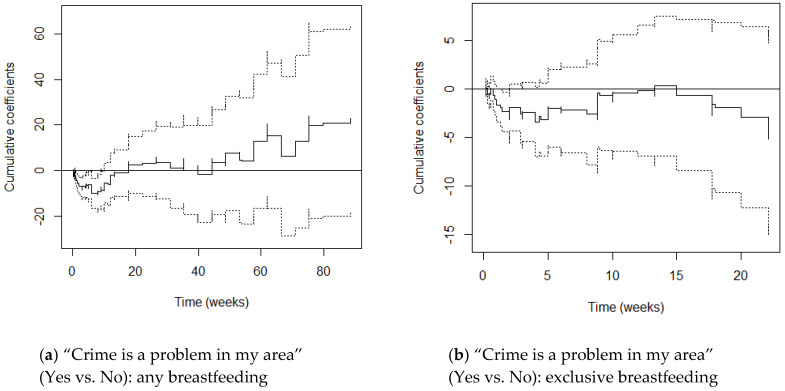
Cox-Aalen model results for “Crime is a problem in my area” (Yes vs. No) for any (**a**) and exclusive breastfeeding (**b**). Main effect set as time-varying, and models adjusted for maternal age and parity (M1). Estimated cumulative coefficients (solid line) with 95% pointwise confidence intervals (dotted lines).

**Figure A3:**
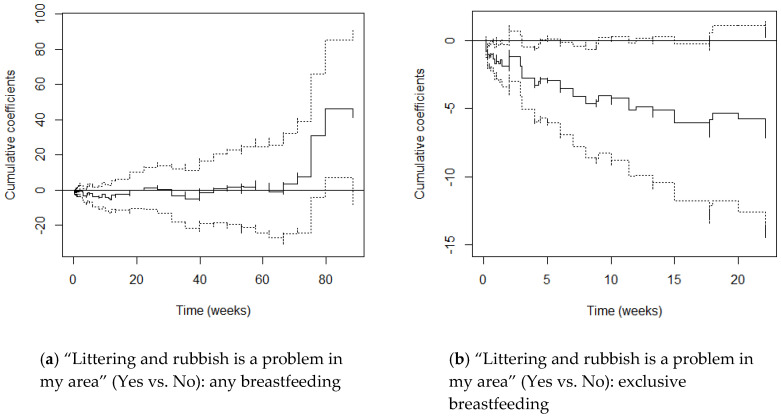
Cox-Aalen model results for “Littering and rubbish is a problem in my area” (Yes vs. No) for any (**a**) and exclusive breastfeeding (**b**). Main effect set as time-varying, and models adjusted for maternal age and parity (M1). Estimated cumulative coefficients (solid line) with 95% pointwise confidence intervals (dotted lines).

**Figure A4:**
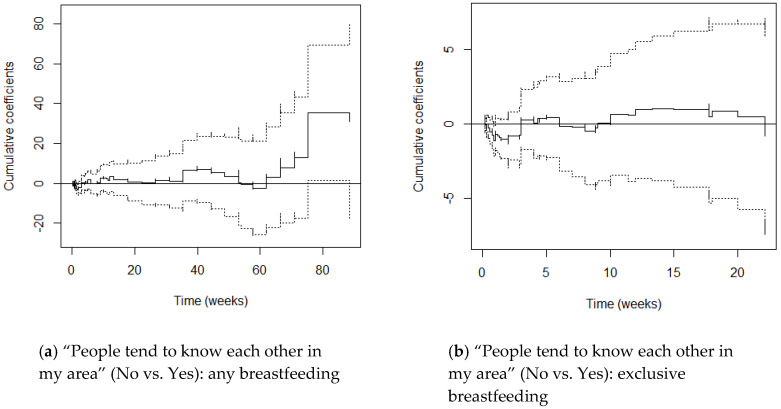
Cox-Aalen model results for “People tend to know each other in my area” (No vs. Yes) for any (**a**) and exclusive breastfeeding (**b**). Main effect set as time-varying, and models adjusted for maternal age and parity (M1). Estimated cumulative coefficients (solid line) with 95% pointwise confidence intervals (dotted lines).

**Figure A5:**
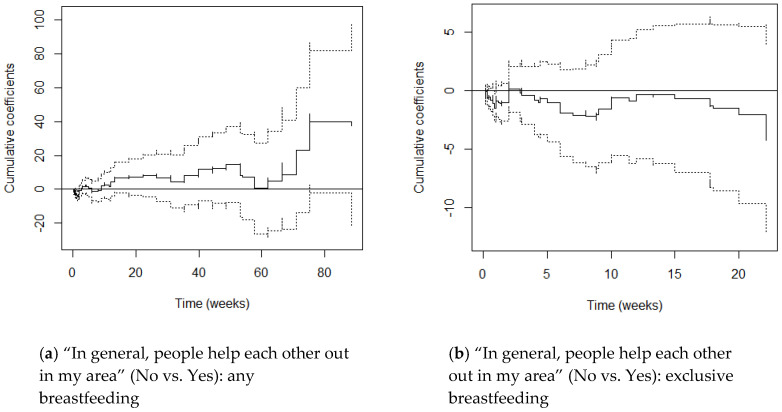
Cox-Aalen model results for “In general, people help each other out in my area” (No vs. Yes) for any (**a**) and exclusive breastfeeding (**b**). Main effect set as time-varying, and models adjusted for maternal age and parity (M1). Estimated cumulative coefficients (solid line) with 95% pointwise confidence intervals (dotted lines).

M1 graphs (adjusting for maternal age and parity) and M2 graphs (adjusting additionally for education) varied little from one another, with cumulative coefficients generally moving just a little more in the negative direction after adjusting for SES. This suggests that once education differentials were accounted for, women’s hazards of stopping breastfeeding reduced only slightly. We focus on the M2 graphs here (Figure 1, Figure 2, Figure 3, Figure 4 and Figure 5) and include the M1 graphs in the Appendix A (Figure A1, Figure A2, Figure A3, Figure A4 and Figure A5). These graphs can be interpreted as solid lines below zero representing a reduced hazard of stopping breastfeeding and solid lines above zero representing an increased hazard. Flat lines represent steady impact over time, lines going downwards represent a declining hazard, lines going upwards represent an increasing hazard. When lines are near zero there is a negligible effect, and where the confidence interval band (dotted lines) includes zero, there is no significant effect of the environmental factor at that point in time.

We now discuss findings for each of the five environmental indicators in turn.

#### 3.2.1. My Area Is a Nice Place to Live

Under the constant effect (i.e., proportional hazards) setting and controlling for maternal age and parity (M1), mothers who disagreed that their area was a nice place to live were 1.44 times as likely to stop any breastfeeding and this reduced to 1.26 times once education was controlled for (M2).

When the proportional hazards assumption was relaxed and Nice was instead set as an additive time-varying effect, we find weak evidence (i.e., at the 10% level) that this indicator had a significant effect on chances of stopping any breastfeeding both before and after adjusting for SES (M1 Supremum-test *p* = 0.069, M2 Supremum-test *p* = 0.066). The Kolmogorov-Smirnov and Cramer von Mises tests did not provide any evidence of time-varying effects. This is confirmed by Figure 1a which has confidence intervals crossing zero throughout. The general trajectory suggests that, albeit non-significantly, compared to mothers who agreed that their area was a nice place to live, those who disagreed generally had increased hazards of stopping breastfeeding but that this fluctuated over time, with reduced hazards observed in the first 5 weeks, between weeks 10 and 15, and in weeks 20–40 (4–9 months).

For exclusive breastfeeding, constant effects were non-significant and only went in the predicted direction before controlling for SES, with mothers who disagreed that their area was a nice place to live having 1.03 times the risk of stopping exclusive breastfeeding compared to those that agreed. The direction of association reversed once education was controlled for, with mothers who disagreed now having *reduced* chances of stopping exclusive breastfeeding (HR 0.86).

When the proportional hazards assumption was relaxed and Nice was set as a time-varying effect, whether mothers thought their area was a nice place to live significantly predicted the chances of stopping exclusively breastfeeding, both before and after controlling for SES (M1 *p* = 0.017, M2 *p* = 0.032). Figure 1b shows that there was a small window within the first week postpartum where mothers who disagreed that their area was a nice place to live had significantly *reduced* hazards of stopping exclusive breastfeeding. Non-significantly reduced hazards were also observed in the first 3 weeks and between 6–13.5 weeks.

#### 3.2.2. Crime Is a Problem in My Area

Under the constant effect setting and controlling for maternal age and parity (M1), mothers who thought that crime was a problem in their area were (non-significantly) 1.15 times as likely to stop any breastfeeding and this reduced to 1.12 times once education was controlled for (M2).

Although there were no significant constant effects, when the proportional hazards assumption was relaxed, Crime was found to significantly predict the chances of stopping any breastfeeding, both before (M1 Supremum-test *p* = 0.006) and after (M2 Supremum-test *p* = 0.002) controlling for SES. However, the high *p*-values of the Kolmogorov-Smirnov and Cramer von Mises score processes tests for constant effects suggested that hazards did not significantly vary over time. However, Figure 2a shows that mothers who thought that crime was a problem in their area had significantly *reduced* hazards of stopping any breastfeeding in the first 10 weeks postpartum. Although confidence intervals cross zero, mothers who considered crime as a problem in their area generally had increased hazards of stopping breastfeeding at later time points.

For exclusive breastfeeding, although constant effects were non-significant they went in the opposite to predicted direction both before and after controlling for SES, with mothers who thought that crime was a problem in their area having *reduced* chances of stopping exclusive breastfeeding (M1 HR 0.91, M2 HR 0.83).

The Supremum-tests suggest that crime did not have a significant effect on exclusive breastfeeding chances when the proportional hazards assumption was relaxed and crime was set as a time-varying effect. The Kolmogorov-Smirnov and Cramer von Mises score processes tests also suggested that hazards did not significantly vary over time. However, Figure 2b shows that mothers who thought that crime was a problem in their area had significantly *reduced* hazards of exclusive breastfeeding between weeks 1 and 2 postpartum. Although confidence intervals cross zero, mothers who considered crime as a problem in their area generally had reduced hazards of stopping exclusive breastfeeding at most time-points.

#### 3.2.3. Littering and Rubbish Is a Problem in My Area

Mothers who thought that littering and rubbish was a problem in their area had a slightly increased but non-significant hazard of stopping any breastfeeding when controlling for age and parity (M1 HR 1.09) and this reduced slightly after controlling for SES (M2 HR 1.05).

Litter was also not significantly associated with stopping any breastfeeding in the non-proportional hazards setting and Kolmogorov-Smirnov and Cramer von Mises score processes tests found no evidence of time-varying effects. 

However, Figure 3a shows that mothers who thought that littering and rubbish was a problem had the predicted increased hazard of stopping any breastfeeding between weeks 53 and 58 (12–13 months) and from week 66 (month 15) onwards, and they were significantly more likely to stop breastfeeding from week 80 (18 months). They did however have almost as much time with *reduced* hazards.

Mothers who thought that littering and rubbish was a problem in their area had *reduced* hazards of stopping exclusive breastfeeding in the constant effects models, and this effect approached significance when SES was controlled for (M2 0.72, *p* = 0.061).

The Supremum-tests suggest that litter had a significant effect on exclusive breastfeeding when its effect was set as time-varying (M1 *p* < 0.001, M2 *p* = 0.002). The high *p*-values of the Kolmogorov-Smirnov and Cramer von Mises score processes tests suggest that hazards did not significantly vary over time but Figure 3b shows a clear time-based effect. The hazards of stopping exclusive breastfeeding declined over time, with mothers who thought that littering and rubbish was a problem having significantly *reduced* hazards at all time-points except between 2 and 3 weeks.

#### 3.2.4. People Tend to Know Each Other in My Area

In the constant effects setting, mothers who reported that people tended not to know each other had non-significantly increased hazards of stopping any breastfeeding (M2 HR 1.16) which increased slightly after adjusting for SES (M2 HR 1.18).

In the time-varying setting, Supremum-tests and Kolmogorov-Smirnov and Cramer von Mises tests showed that whether mothers reported that people knew each other had no significant effect and no time-varying effect on any breastfeeding, respectively. However, inspection of Figure 4a shows that these mothers had *reduced* hazards of stopping any breastfeeding in the first 5 weeks (significantly so around week 2), at 6–8 weeks and between weeks 58 and 62 (13–14 months). Their hazards were otherwise generally increased, and significantly so from week 75 (17 months), compared to mothers who thought that people tended to know each other.

When the effects were set as constant, mothers who thought that people tended not to know each other had non-significantly *reduced* hazards of stopping exclusive breastfeeding (M1 HR 0.90, M2 HR 0.87). When effects were set as time-varying, we found no evidence of a significant or time-varying effect on exclusive breastfeeding. However, Figure 4b shows that hazards of stopping exclusive breastfeeding were generally *reduced* and significantly so for a few days around 1 week postpartum; hazards were only increased around weeks 3–4, 4.5–6 weeks, 10–11.5 weeks, and weeks 12–17.5.

#### 3.2.5. In General, People Help Each Other in My Area

Whether mothers thought that people helped each other out in their area did not significantly predict chances of any breastfeeding in the constant effect setting, although associations were in the predicted direction (M1 HR 1.31, M2 HR 1.23).

Supremum-tests and Kolmogorov-Smirnov and Cramer von Mises tests showed that whether mothers thought that people helped each other had no significant effect and no time-varying effect on any breastfeeding, respectively. Figure 5a does however show that mothers who thought that people helped each other out in their area had significantly *reduced* hazards of stopping any breastfeeding in the first 2 weeks. Hazards started to be increased (albeit non-significantly) from around week 10, staying increased for the remainder of women’s infant feeding journeys except during weeks 58–62 (13–14 months). Although the increased hazards were non-significant, the lower confidence band approaches zero from 75 weeks (17 months).

Whether mothers thought that people helped each other out was associated with *reduced* hazards of stopping exclusive breastfeeding in the constant effects models, approaching significance once SES was controlled for (M2 HR 0.65, *p* = 0.081).

Supremum-tests and Kolmogorov-Smirnov and Cramer von Mises tests showed no significant or time-varying effects in the non-proportional hazards setting. However, Figure 5b shows hazards of stopping exclusive breastfeeding were consistently reduced for mothers who thought that people did not help each other out, with a significant reduction seen for a few days around 1 week postpartum.

Overall, these findings suggest that generally the risks of stopping any breastfeeding increase with time (but after a while) and the risks of stopping exclusive breastfeeding decrease steadily over time.

## 4. Discussion

Given the literature on environmental effects on parenting, reproduction and health, we would expect mothers to stop breastfeeding sooner when they perceive their environments less favourably. We found limited support for this hypothesis. Women’s hazards of stopping both any and exclusive breastfeeding varied over time, with subjective environmental experiences only being associated with breastfeeding chances at specific timepoints, and not always in the predicted direction. Whilst results were not necessarily significant according to the conventional alpha cut-off of 0.05, consistent trends highlight that real effects may exist and are important to consider [58,59].

Our findings suggest that the first few weeks postpartum were most susceptible to environmental influences and that contrary to our predictions, mothers with worse subjective environmental experiences were *less* likely to stop breastfeeding at this time. Mothers who thought that littering and rubbish was a problem in their area also showed a *declining* hazard of stopping exclusive breastfeeding over time. The predicted increased hazard of stopping breastfeeding was only evident in the later stages of any breastfeeding and only for mothers who reported littering as a problem or that people tended not to know each other. We now discuss each of these key findings in turn before considering study limitations and proposing ideas for future research.

The reduced hazards of stopping breastfeeding in the first few weeks postpartum were evident for all five of our environmental indicators to varying degrees, with mothers who thought that crime was a problem in their area having the most pronounced effects. Most crime and health research focuses on the health consequences of directly experiencing crime; victims of violent crime suffer a range of health problems. The initial acute physical injury, increased stress and increased risk of mental health problems can lead to chronic physical injury, impaired immune system functioning, increased health risk behaviour and inappropriate health care utilisation, all resulting in further increased risks of health problems [60]. Research explicitly linking crime and breastfeeding is largely limited to the effects of experiencing domestic violence; the evidence is inconclusive but suggests that many mothers who have experienced abuse may be less likely to breastfeed [52,53,54,55]. Direct experience of crime and domestic violence may have negative health impacts and reduce women’s breastfeeding chances, but what about crime perception and crime outside the home?

Just as agreement between objective and subjective measures of environmental quality tends to be only low to moderate [32], fear of crime is not just a response to high crime rates with the two tending to be only weakly correlated [39]. This apparent disentangling of subjective and objective assessments of crime seems to be driven by individual factors. For example, women tend to fear crime to a greater extent than men (and to fear different types of crime). Furthermore, vicarious fear expressed by husbands or partners may lead to restrictions on women’s activities [39]. Other factors influencing fear of crime are previous direct experience of crime, mental health, ethnicity, and age [39]. Whilst we had data on these last two factors, the vast majority of our sample of mothers were White and so fears specifically related to racially motivated crimes are unlikely to be relevant for this sample.

Regardless of the extent to which it correlates with objective crime statistics or other individual factors, fear of crime has been to shown to have its own negative health impacts [39,61]. Pleasant neighbourhoods are associated with better wellbeing and feelings of safety whereas unsafe neighbourhoods may discourage social interaction [39], potentially negatively impacting breastfeeding which tends to be more successful with adequate social support [43,62,63,64]. Furthermore, social norms discouraging breastfeeding may prevail in disadvantaged environments [65], so any social interaction that does occur may reduce breastfeeding chances.

Fear of crime can lead to avoidance behaviours including self-isolation [61] thus we can speculate that new mothers scared of crime may feel safer staying at home, and staying at home in the first few weeks postpartum may help with infant bonding and establishing successful breastfeeding [66]. Avoidance behaviours coupled with the protective effect of “nesting in” with a newborn may also explain the reduced hazards of stopping breastfeeding seen for some of the other environmental experience measures, such as whether mothers thought their area was a nice place to live or whether littering and rubbish was a problem, which both conferred reduced hazards of stopping exclusive breastfeeding early on. Of course if mothers are experiencing breastfeeding difficulties, then they may benefit from seeking social support [4], but friends, family and health professionals could make home visits if mothers did not want to leave the house, and many services are accessible on the phone or via the internet. The reduced hazards of stopping breastfeeding in the early weeks for those mothers that report that people do not tend to know or help each other in their area does however suggest that this kind of social support, whether or not provided at home, may be coming from people outside the areas where mothers live. This seems plausible given that it is common for mothers, especially those of higher SES, to live far away from their own parents [67].

Our questionnaire asked mothers whether they thought crime was a problem in their area, which perhaps may not be addressing fear of crime itself. Mothers may have thought crime was a problem without being scared to go out and we do not know which crimes they were considering as problematic. Similarly, the type and extent of littering and rubbish is unknown, but we can imagine that in this relatively high SES sample, the kinds of crimes, littering and rubbish considered to be problematic are likely to differ from those in more deprived areas. Avoidance behaviour may however only makes sense in extreme cases of environmental contamination and could be a less likely explanation for the persistently declining hazard of stopping exclusive breastfeeding seen amongst mothers who considered littering and rubbish to be a problem. What then might explain this finding?

Given that the trajectories of any and exclusive breastfeeding differ so greatly for mothers’ perceptions of littering and rubbish, with the former showing no association except in the later stages of breastfeeding and the latter showing a clear reduction in hazards over time, we can speculate that there may be important differences between mothers who breastfeed exclusively and those who combine breastfeeding with formula. The exclusive breastfeeding models remove mothers who used mixed methods of infant feeding, and so the differently shaped graphs could suggest that mixed-feeding mothers’ breastfeeding chances were either unaffected by their perception of littering, or that they were more likely to stop any breastfeeding when they perceived littering and rubbish as a problem. In other words, formula feeding may be confounding the association. This could mean that, for example, littering and rubbish may be more of a deterrent to formula feeding in environments perceived as dirty, with mothers concerned that there is nowhere clean to prepare formula. Less deprived areas, which likely correspond with being a nicer place to live, with less litter, crime etc., tend to have more “mummy cafes” with facilities for preparing formula feeds as well as being a more friendly place to breastfeed. On the other hand, in more deprived areas, beyond supermarket cafes and big chain restaurants, facilities for formula feeding are lacking. Breastfeeding may therefore be perceived as more convenient [68], although mothers may be more concerned that they have nowhere sanitary to sit whilst breastfeeding [69,70]. Another potential explanation is that the littering and rubbish question may be acting as a proxy for other aspects of the environment, which may be behind the observed associations. For example, when considered as a physical incivility, littering may be signaling antisocial behaviour, which may explain why it has been found to correlate with increased fear of crime in other studies [39]. Littering and rubbish may also correspond with other physical environmental issues such as levels of air and water pollution and together these broader environmental quality concerns may be driving women to stay home and avoid these exposures [10]. Alternatively, as breastfeeding provides antioxidative protection against some of the detrimental health impacts of prenatal environmental contaminant exposure [71,72,73,74], mothers who are aware of these benefits may make a conscious decision to continue breastfeeding to protect their infants from environmental harm. It is helpful to think through what might explain the observed associations but these are of course just speculations that go beyond the capabilities of our quantitative data; qualitative research is needed to unpack some of the underlying mechanisms and to understand how these environmental factors play out alongside sociocultural, policy and marketing drivers.

Whilst littering and rubbish was associated with reduced hazards of stopping exclusive breastfeeding, it was associated with increased hazards of stopping any breastfeeding from around week 75 (17 months). Whilst mothers are more likely to give up breastfeeding in the early weeks if they’re struggling, logically they are increasingly likely to stop breastfeeding as time goes on, as they transition to introducing their infants to complementary foods. It may just take one small push for a mother to stop breastfeeding at this stage, and perhaps a (perceived) dirty environment is enough for a wavering mother to stop. A perceived lack of neighbourhood friendliness and support (as indexed by whether mothers thought people tended to know or help each other in their area) may similarly be enough to push mothers to stop breastfeeding at the later stages. In the UK, breastfeeding outside the home is still difficult for many mothers [75,76] and negative reactions from the public are common [77], especially if the child is older [78]. Perceiving others as unkind or unhelpful may exacerbate women’s concerns over breastfeeding outside the home.

### Limitations and Recommendations for Further Research

There are many factors that affect women’s infant feeding decisions. This survey was conducted to primarily explore the role of social support in shaping women’s infant feeding experiences and some of our other research using this dataset explores this further [43]. This paper presents a secondary analysis of the data exploring different drivers of infant feeding behaviour but does not preclude the role of other important sociocultural drivers and contextual factors.

Our analytical sample size combined with limited variation in exposures and outcomes means we are only likely to identify medium and large effect sizes at *p* ≤ 0.05. Given the relatively consistent direction of effect (within any and exclusive breastfeeding), our null results in the constant effect (proportional hazards) models may suggest a power issue. A larger sample size may therefore mitigate against the risk of a Type II error. However, as the cumulative coefficient graphs suggested, perhaps relationships between environmental factors and breastfeeding outcomes are better described by time-varying effects.

In addition, our results are unlikely to be representative. Mothers were recruited online, primarily via Facebook groups which have biased our sample towards higher SES individuals. Studies have shown that social media survey recruitment can lead to an increased proportion of middle-class participants; however, this trend is not consistent, and it can still be an effective way to enroll “hard-to-reach” populations [79], especially through tailored and targeted recruitment (for further discussion see [43]). However, the high SES bias in our sample is evident in the descriptive statistics in Table 1, where education and annual income levels are far greater than country averages for the same time period [80,81]. Our findings are therefore only exploratory and descriptive in nature and not generalizable to the broader general UK population of new mothers; larger, more representative samples are needed to verify our results. Further research could replicate this study with more representative samples with more diverse SES and environmental experiences, including in non-WEIRD (Western, Educated, Industrialized, Rich and Democratic) populations [82].

We do not know what women were doing in the “Any breastfeeding” category other than that they were giving their infants some breastmilk, so future research, including using qualitative methodology, could look at whether subjective environmental experiences predict patterns of feeding behaviour within this group. It would also be interesting to explore how subjective environmental experiences map onto objective measures of environmental quality to see how accurate women’s risk perceptions are. For example, how mothers’ crime perception maps onto local crime statistics. This was not possible in the current study as we only had the region in which mothers lived; future studies could collect postcode information to match up smaller scale geographical information with women’s subjective environmental experiences. Given that SES has been shown to buffer against environmental effects on breastfeeding [9], further research could also explore how women’s subjective environmental experiences interact with socioeconomic status and other individual characteristics such as ethnicity (and prior victimization in the case of crime) to impact breastfeeding chances (we attempted to explore interactions between SES and the five environmental measures in this study but small numbers in combinatorial categories prevented the models from converging. This kind of analysis would require larger sample sizes). There is also a need for more qualitative research to explore the role of environmental perception in women’s infant feeding decisions further. Possible questions to address include: What crimes are perceived as a problem? Does littering and rubbish lead to avoidance behaviour? Do women recognise these as threatening breastfeeding? Or are there other indirect links to breastfeeding outcomes?

## 5. Conclusions

Our study has highlighted that even in this relatively homogenous high SES sample, women’s hazards of stopping breastfeeding were not constant over time and there were particular timepoints in their breastfeeding journeys where subjective environmental experiences may have played a role. In particular we found that contrary to our predictions, women with worse subjective environmental experiences were *less* likely to stop breastfeeding in the first few weeks postpartum, especially women who perceived crime as a problem in their area. We also found that women who thought that littering and rubbish was a problem in their area were progressively *less and less* likely to stop exclusive breastfeeding. Lastly, the increased hazards from around 75 weeks (17 months) onwards for mothers who perceived littering and rubbish as a problem or who thought that people tended not to know each other was the only evidence we found in support of our prediction that mothers who perceived their environments unfavourably would have lower breastfeeding chances.

Further research with larger, more diverse and representative samples is warranted to explore these associations further. It would also be helpful to conduct qualitative research to understand whether mothers deliberately alter their infant feeding behavior in response to environmental threats or whether other mechanisms are at play.

## Figures and Tables

**Table 1:** Characteristics of respondents and their association with breastfeeding outcomes.

Characteristics		Breastfeeding Initiation (*n* = 512)		Duration of Any Breastfeeding (weeks) (*n* = 480)			Duration of Exclusive Breastfeeding (weeks) (*n* = 293)		
	*n*	*n* (%)	*p*-Value ^1^	*n*	Median (IQR)	*p*-Value ^3^	n	Median (IQR)	*p*-Value ^3^
**Subjective environmental experiences**									
***My area is a nice place to live***									
Yes	482	453 (94.0%)		453	31.7 (41.7)		277	22.1 (23.7)	
No	22	20 (90.9%)	0.638	20	19.0 (34.3)	0.113	13	13.3 (24.6)	0.630
Missing	8	7 (87.5%)		7	53.4 (49.6)		3	26.6 (0.0)	
***Crime is a problem in my area***									
No	378	355 (93.9%)		355	31.0 (42.4)		213	22.1 (24.6)	
Yes	59	54 (91.5%)	0.564	54	33.8 (44.2)	0.331	37	22.1 (20.6)	0.537
Missing	75	71 (94.7%)		71	31.7 (44.3)		43	17.7 (23.6)	
***Littering and rubbish is a problem in my area***									
No	387	364 (94.1%)		364	31.4 (43.0)		220	22.1 (24.6)	
Yes	104	97 (93.3%)	0.947 ^2^	97	29.4 (50.0)	0.486	63	22.1 (19.6)	0.179
Missing	21	19 (90.5%)		19	34.0 (20.2)		10	24.4 (8.3)	
***People tend to know each other in my area***									
Yes	329	308 (93.6%)		308	34.3 (43.1)		186	22.1 (24.4)	
No	121	116 (95.9%)	0.470 ^2^	116	27.6 (42.0)	0.313	74	20.1 (23.6)	0.882
Missing	62	56 (90.3%)		56	32.4 (40.6)		33	26.6 (21.6)	
***In general, people help each other out in my area***									
Yes	361	339 (93.9%)		339	33.0 (41.4)		205	22.1 (23.7)	
No	77	71 (92.2%)	0.607	71	28.0 (40.9)	0.419	46	26.6 (24.3)	0.596
Missing	74	70 (94.6%)		70	27.2 (52.4)		42	17.7 (23.3)	
**Area**									
***England***									
East Midlands	21	16 (76.2%)		16	27.4 (39.1)		12	26.6 (17.8)	
East of England	34	31 (91.2%)		31	31.0 (27.6)		18	26.6 (19.6)	
South East	89	83 (93.3%)		83	29.4 (51.1)		51	17.7 (25.6)	
Yorkshire & The Humber	57	54 (94.7%)		54	35.5 (40.7)		36	26.6 (20.6)	
North East	45	43 (95.6%)		43	26.3 (29.2)		20	13.3 (25.2)	
West Midlands	23	22 (95.7%)		22	16.5 (40.6)		11	3.0 (16.0)	
London	69	66 (95.7%)		66	35.4 (37.0)		46	24.4 (19.1)	
North West	61	59 (96.7%)		59	35.4 (46.6)		38	21.1 (22.3)	
South West	54	53 (98.1%)		53	29.9 (42.7)		26	19.9 (23.3)	
***Wales***	21	18 (85.7%)		18	33.2 (26.8)		12	16.3 (22.8)	
***Scotland***	32	29 (90.6%)		29	30.3 (32.1)		20	22.1 (23.8)	
***Northern Ireland***	6	6 (100.0%)	0.607	6	33.2 (44.3)	0.926	3	0.9 (13.1)	0.439
**Maternal and infant characteristics**									
***Maternal age (years)***									
16–24	24	20 (83.3%)		20	24.4 (38.7)		9	6.0 (25.1)	
25–29	98	87 (88.8%)		87	22.1 (44.1)		55	12.0 (25.1)	
30–34	245	232 (94.7%)		232	34.5 (46.5)		140	22.1 (23.9)	
35–39	121	119 (98.3%)		119	35.4 (34.6)		76	26.6 (16.9)	
40–44	24	22 (91.7%)	0.005	22	27.4 (44.5)	0.194	13	26.6 (25.6)	0.150
***Parity***									
1st birth	313	292 (93.3%)		292	31.0 (41.7)		177	18.0 (24.6)	
2nd or higher	199	188 (94.5%)	0.726 ^2^	188	32.6 (44.5)	0.921	116	26.6 (22.1)	0.083
***Ethnicity***									
White	490	459 (93.7%)		459	31.7 (43.1)		282	22.1 (23.6)	
Other	22	21 (95.5%)	1.000	21	29.4 (44.1)	0.710	11	17.1 (25.4)	0.724
***Partnership status***									
Partnered	501	469 (93.6%)		469	31.1 (43.1)		288	22.1 (23.6)	
Unpartnered	11	11 (100.0%)	1.000	11	52.6 (41.9)	0.295	5	26.6 (25.6)	0.993
***Birthweight***									
Normal (≥2500 g)	483	452 (93.6%)		452	31.9 (43.2)		282	22.1 (23.6)	
Low (<2500 g)	20	20 (100.0%)	0.626	20	28.1 (33.7)	0.609	8	13.4 (25.5)	0.561
Missing	9	8 (88.9%)		8	13.4 (21.7)		3	8.9 (8.4)	
**Socioeconomic Status**									
***Education***									
GCSEs or AS/A-levels	94	84 (89.4%)		84	19.5 (36.5)		56	8.0 (25.1)	
Graduate	198	179 (90.4%)		179	27.9 (35.9)		107	26.6 (23.6)	
Postgraduate	220	217 (98.6%)	<0.001 ^2^	217	38.7 (41.0)	<0.001 ^4^	130	26.3 (22.6)	0.003 ^4^
***Annual income***									
Under £50,000	206	189 (91.7%)		189	31.7 (44.3)		113	26.6 (23.7)	
£50,000 plus	278	264 (95.0%)	0.215 ^2^	264	31.0 (39.9)	0.782	159	22.1 (23.6)	0.472
Missing	28	27 (96.4%)		27	38.9 (42.1)		21	17.7 (18.6)	
***Financial Situation***									
Finding it difficult	34	30 (88.2%)		30	22.1 (42.5)		14	7.4 (24.6)	
Just about getting by	100	96 (96.0%)		96	31.9 (43.8)		58	26.6 (21.3)	
Doing alright	230	216 (93.9%)		216	31.0 (42.4)		136	22.1 (24.6)	
Living comfortably	144	134 (93.1%)	0.408	134	35.4 (41.6)	0.643	81	22.1 (22.6)	0.559
Missing	4	4 (100.0%)		4	17.7 (11.2)		4	17.7 (5.6)	
***Partner’s employment status***									
Unemployed	12	10 (83.3%)		10	36.6 (77.6)		8	26.6 (20.2)	
Employed	489	459 (93.9%)	0.175	459	31.1 (42.9)	0.440	280	22.1 (23.6)	0.503
Missing	11	11 (100.0%)		11	52.6 (41.9)		5	26.6 (25.6)	

^1^ Fisher’s Exact Test unless otherwise specified. ^2^ Pearson’s Chi-squared test with Yates’ continuity correction. ^3^ Wilcoxon rank test used for binary variables and Kruskal-Wallis used when 3 or more categories. ^4^ Post-hoc Dunn tests showed only GCSEs or AS/A-levels vs. Graduate (*p* < 0.001) and Graduate vs. Postgraduate (*p* = 0.006) had significantly different any breastfeeding median durations and only GCSEs or AS/A-levels vs. Graduate (*p* = 0.004) and GCSEs or AS/A-levels vs. Postgraduate (*p* = 0.003) had significantly different exclusive breastfeeding median durations (*p*-values adjusted with the Benjamini-Hochberg method for multiple comparisons).

**Table 2:** Constant and time-varying effects of subjective environmental experience on duration of any breastfeeding.

		M1: Controlling for Age and Parity					M2: Additionally Controlling for Education				
		Constant Effect		Time-Varying Effect			Constant Effect		Time-Varying Effect		
	*n*	HR (95% CI)	*p*-Value	Sup. *p*-Value	K-S *p*-Value	CvM *p*-Value	HR (95% CI)	*p*-Value	Sup. *p*-Value	K-S *p*-Value	CvM *p*-Value
**Nice** **(No vs. Yes)**	473	1.44 (0.73–2.86)	0.351	0.069	0.480	0.617	1.26 (0.62–2.53)	0.569	0.066	0.455	0.607
**Crime** **(Yes vs. No)**	409	1.15 (0.75–1.77)	0.551	0.006	0.755	0.676	1.12 (0.72–1.74)	0.642	0.002	0.590	0.470
**Litter** **(Yes vs. No)**	461	1.08 (0.76–1.53)	0.693	0.139	0.179	0.156	1.05 (0.74–1.51)	0.789	0.114	0.256	0.132
**Know** **(No vs. Yes)**	424	1.16 (0.82–1.63)	0.441	0.694	0.331	0.364	1.18 (0.84–1.67)	0.391	0.631	0.328	0.432
**Help** **(No vs. Yes)**	410	1.31 (0.89–1.95)	0.239	0.593	0.387	0.54	1.23 (0.82–1.82)	0.408	0.385	0.520	0.682

Cox-Aalen model results for duration of any breastfeeding. HR: Hazard Ratio. 95% CI: 95% Confidence Interval). Sup. *p*-values: Supremum-test *p*-values testing the significance of parameters under the non-proportional hazards setting. K-S *p*-values and CvM *p*-values: Kolmogorov-Smirnov test and Cramer von Mises test *p*-values for score processes testing if effects are non-proportional (H_0_ = constant effect). Nice: “My area is a nice place to live”. Crime: “Crime is a problem in my area”. Litter: “Littering and rubbish is a problem in my area”. Know: “People tend to know each other in my area”. Help: “In general, people help each other out in my area”.

**Table 3:** Constant and time-varying effects of subjective environmental experience on duration of exclusive breastfeeding.

		M1: Controlling for Age and Parity					M2: Additionally Controlling for Education				
		Constant Effect		Time-Varying Effect			Constant Effect		Time-Varying Effect		
	*n*	HR (95% CI)	*p*-Value	Sup. *p*-Value	K-S *p*-Value	CvM *p*-Value	HR (95% CI)	*p*-Value	Sup. *p*-Value	K-S *p*-Value	CvM *p*-Value
**Nice** **(No vs. Yes)**	290	1.03 (0.49–2.19)	0.928	0.017	0.587	0.851	0.86 (0.40–1.85)	0.692	0.032	0.682	0.865
**Crime** **(Yes vs. No)**	250	0.91 (0.55–1.49)	0.662	0.139	0.458	0.369	0.83 (0.50–1.40)	0.431	0.131	0.463	0.383
**Litter** **(Yes vs. No)**	283	0.78 (0.52–1.17)	0.158	<0.001	0.434	0.227	0.72 (0.47–1.09)	0.061	0.002	0.374	0.163
**Know** **(No vs. Yes)**	260	0.90 (0.62–1.32)	0.584	0.818	0.351	0.247	0.87 (0.59–1.27)	0.458	0.692	0.637	0.271
**Help** **(No vs. Yes)**	251	0.77 (0.48–1.23)	0.266	0.159	0.236	0.127	0.65 (0.40–1.05)	0.081	0.202	0.207	0.146

Cox-Aalen model results for duration of exclusive breastfeeding. HR: Hazard Ratio. 95% CI: 95% Confidence Interval). Sup. *p*-values: Supremum-test *p*-values testing the significance of parameters under the non-proportional hazards setting. K-S *p*-values and CvM *p*-values: Kolmogorov-Smirnov test and Cramer von Mises test *p*-values for score processes testing if effects are non-proportional (H_0_ = constant effect). Nice: “My area is a nice place to live”. Crime: “Crime is a problem in my area”. Litter: “Littering and rubbish is a problem in my area”. Know: “People tend to know each other in my area”. Help: “In general, people help each other out in my area”.

## Appendix A

**Table A1:** Cox and Aalen model diagnostics testing for proportional hazards assumption and time invariant effects to determine whether to set covariate effects as time-varying in main models.

Covariates	Any Breastfeeding (*n* = 480)							Exclusive Breastfeeding (*n* = 293)						
	Cox Model: Test for PH			Aalen Model: Tests for Time-Invariant Effects				Cox Model: Test for PH			Aalen Model: Tests for Time-Invariant Effects			
	rho	Chi^2^	*p*-Value	K-S Test	K-S *p*-Value	C Test	C *p*-Value	rho	Chi^2^	*p*-Value	K-S test	K-S *p*-Value	C Test	C *p*-Value
**Maternal age (years): ref. = 16–24**														
25–29	0.143	9.010	0.003	114.0	0.001	365,000.0	<0.001	0.004	0.003	0.954	13.5	0.173	769.0	0.238
30–34	0.152	11.490	0.001	112.0	<0.001	419,000.0	<0.001	0.020	0.085	0.771	21.0	0.026	2240.0	0.030
35–39	0.177	20.470	<0.001	121.0	<0.001	438,000.0	<0.001	0.027	0.248	0.618	24.2	0.008	3310.0	0.007
40–44	0.152	11.070	0.001	135.0	0.001	596,000.0	<0.001	−0.130	3.541	0.060	46.7	<0.001	18,300.0	<0.001
**Parity: ref. = 1st birth**														
2nd +	−0.043	0.190	0.663	18.4	0.452	6500.0	0.396	0.027	0.145	0.704	3.5	0.241	54.1	0.313
**Education: ref: = GCSEs or AS/A–levels**														
Graduate	0.177	14.610	<0.001	43.2	0.078	56,000.0	0.044	−0.014	0.031	0.861	4.4	0.244	154.0	0.105
Postgrad	0.125	5.690	0.017	39.9	0.029	60,000.0	0.003	0.044	0.493	0.483	4.0	0.280	94.6	0.197

PH: Proportional Hazards. K-S: Kolmogorov-Smirnov. CvM: Cramer von Mises test. *p*-Values below 0.05 indicate evidence against the null of proportional hazards and time-invariant effects. Covariates were considered non-proportional/time-varying where at least two of the three tests provided evidence against the null for the majority of the covariate’s categories.
